# Comparison of clinical utilities of the platelet count and platelet-lymphocyte ratio for predicting survival in patients with cervical cancer: a single institutional study and literature review

**DOI:** 10.18632/oncotarget.19560

**Published:** 2017-07-25

**Authors:** Katsumi Kozasa, Seiji Mabuchi, Naoko Komura, Eriko Yokoi, Kuroda Hiromasa, Tomoyuki Sasano, Mahiru Kawano, Yuri Matsumoto, Eiji Kobayashi, Tadashi Kimura

**Affiliations:** ^1^ Departments of Obstetrics and Gynecology, Osaka University Graduate School of Medicine, Suita, Osaka, Japan

**Keywords:** cervical cancer, thrombocytosis, survival, platelet count, platelet-lymphocyte ratio

## Abstract

**Objective:**

To compare the clinical utilities of the platelet count and platelet-lymphocyte ratio (PLR) for predicting survival in patients with cervical cancer.

**Results:**

Multivariate analyses demonstrated that thrombocytosis and elevated PLR were found to be independent prognostic factors for progression-free survival (PFS, *P* = 0.0077, *P* = 0.044) and overall survival (OS, *P* = 0.025, *P* = 0.019) in separate Multivariate analyses. In the ROC analysis, the platelet count showed a significantly greater area under the ROC curve (AUC) value than that of PLR for predicting patient recurrence (0.5941 versus 0.5331, *p* = 0.018) and survival (0.6139 versus 0.5468, *p* = 0.029). In patients without thrombocytosis, elevated PLR correlated with shorter survival (PFS, *P* = 0.041; OS, *P* = 0.017). In contrast, PLR in patients with thrombocytosis did not provide prognostic information. We divided patients into 3 prognostic groups using platelet counts and PLR: high-risk (thrombocytosis with any PLR); intermediate-risk (elevated PLR without thrombocytosis); low-risk (none of the above), which allowed for individualized and accurate survival estimates.

**Materials and Methods:**

The baseline characteristics and clinical outcomes of cervical cancer patients were identified. Patients were grouped according to their pretreatment platelet counts or PLR, and clinicopathological characteristics and patient survival were then compared between these groups. The clinical utilities of the platelet count and PLR were compared using a time-dependent receiver operating characteristic (ROC) analysis.

**Conclusions:**

Pretreatment thrombocytosis and elevated PLR were identified as independent predictors in cervical cancer patients. Platelet counts were superior to PLR for predicting the prognosis of uterine cervical cancer patients. Our prognostic model consisting of platelet counts and PLR offers individualized survival estimates.

## INTRODUCTION

Cervical cancer is the second most common type of cancer affecting women worldwide and has an annual incidence of 530,000 new cases. Although current standard treatments for invasive cervical cancer are potentially curative, a significant number of patients develop recurrence and die of disease progression, with approximately 250,000 deaths being reported globally each year [1].

The identification of new prognostic factors for cervical cancer will improve our understanding of cervical cancer biology, contribute to the stratification of patients into risk groups, and identify those at a high risk of recurrence after the standard initial treatment.

Platelet count alterations including the platelet count and platelet-lymphocyte ratio (PLR) have recently been attracting attention as prognostic indicators in cancer patients [2–18]. The relationship between elevated platelet counts and malignancy was initially described in 1872 [19]. Since then, an increasing number of studies have reported thrombocytosis in patients with cancer from various origins, and demonstrated that it is associated with poor patient prognosis [20–23]. To the best of our knowledge, 13 studies have investigated the prognostic implications of thrombocytosis in cervical cancer patients, with about half suggesting that thrombocytosis is an independent prognostic factor in cervical patients (Table 1). A recent study on ovarian cancer indicated that paraneoplastic thrombocytosis is due to the enhancements induced in hepatic thrombopoietin synthesis by tumor-derived IL-6. Moreover, the inhibition of thrombopoietin and IL-6 expression abrogated thrombocytosis in tumor-bearing mice and significantly enhanced the therapeutic efficacy of paclitaxel in mouse models of epithelial ovarian cancer [24]. Thus, thrombocytosis is now regarded not only as a prognostic indicator, but also as a potential therapeutic target in human cancers.

**Table 1:** Summary of studies that investigated the relationship between platelet counts, platelet-lymphocyte ratios, and survival in patients with cervical cancer

Reference	No.	Stage	Treatment	Platelet or PLR	Cut-off value	Results	Multivariate analysis
Hernandez et al. [2], 1992	113	I-IV	RT	Platelet	400 × 10^3^/μl	Independent prognostic indicator of 5-year survival	Yes
Rodriguez et al. [3], 1994	219	IB	Surgery	Platelet	300 × 10^3^/μl	Independent prognostic indicator of 5-year survival	Yes
Hernandez et al. [4],1994	623	IB	Surgery	Platelet	400 × 10^3^/μl	Not independent prognostic indicator of 5-year survival	Yes
Lopes et al. [5], 1994	643	I-IV	Surgery or RT	Platelet	400 × 10^3^/μl	Prognostic indicator of 5-year survival	No
De Jonge et al. [6], 1999	93	IB	Surgery	Platelet	400 × 10^3^/μl	Not independent prognostic indicator of 5-year survival	yes
Hernandez et al. [7],2000	291	IIB-IVA	RT	Platelet	400 × 10^3^/μl	Independent prognostic indicator of OS (patients negative pelvic nodes)	Yes
Gadducci et al. [8], 2010	46	IB2-IIB	Surgery	Platelet	272 × 10^3^/μl	Independent prognostic indicator of OS, but not of PFS	Yes
Gadducci et al. [9], 2010	140	IB2-IIB	Surgery	Platelet	270 × 10^3^/μl	Not independent prognostic indicator of PFS and OS	Yes
Qiu et al. [10], 2010	318	I-IV	NA	Platelet	400 × 10^3^/μl	Not prognostic indicator of OS	No
Wang et al. [11], 2012	111	IB2-IIB	Surgery	Platelet	266 × 10^3^/μl	Not prognostic indicator of PFS and OS	No
Zhang et al. [12],2014	460	I-II	Surgery	PLR	150	Not prognostic indicator of PFS and OS	No
Kawano et al. [13], 2015	286	IB-IVA	RT	Platelet	350 × 10^3^/μl	Independent prognostic indicator of OS	Yes
Xiao et al. [14], 2015	238	I-IV	CCRT	Platelet	200 × 10^3^/μl	Not prognostic indicator of PFS and OS	No
Zhao et al. [15], 2015	220	I-IIA	Surgery	Platelet	300 × 10^3^/μl	Not independent prognostic indicator of OS	Yes
Nakamura et al. [16], 2015	32	NA	CCRT	PLR	322	Independent prognostic indicator of 200-day survival	Yes
Zheng et al. [17], 2016	795	IA-IIA	Surgery	PLR	128.3	Independent prognostic indicator of OS	Yes
Chen et al. [18], 2016	407	IB-IIA	Surgery	PLR	138.35 (PFS), 143.47 (OS)	Independent prognostic indicator of PFS and OS	Yes
Present study, 2017	684	IA-IVA	Surgery or RT	Platelet, PLR	350 × 10^3^/μl (Platelet), 125.23 (PFS), 131.44 (OS)	Both factors are independent prognostic indicator of PFS and OS, Predictive value of platelet count is greater than that of PLR	Yes

RT, radiotherapy; CCRT, concurrent chemoradiotherapy; PLR, platelet-lymphocyte ratio; PFS, progression free survival; OS, overall survival; NA, not available.

As shown in Table 1, 4 studies have investigated the prognostic implications of increased PLR in cervical cancer patients, with 3 studies suggesting that increased PLR is an independent predictor of survival. However, since most of the studies described above only included surgically-treated early-stage cervical cancer patients, the prognostic significance of PLR in cervical cancer remains unclear. Moreover, the clinical utilities of platelet counts and PLR have not yet been compared, and there is currently no information on how physicians may distinguish between platelet counts and PLR in the management of cervical cancer.

**Table 2:** Clinicopathological characteristics of patients according to platelet counts and PLR ^1^

		All patients		Thrombocytosis		Normal platelet count			Elevated PLR		Normal PLR		
		No	(%)	No	(%)	No	(%)	*p*-value	No	(%)	No	(%)	*p*-value
Age	< 50	268	39.2	52	19.4	216	80.6		123	45.9	145	54.1	
	≥ 50	416	60.8	35	8.4	381	91.6	< 0.001	140	33.7	276	66.3	0.0013
Stage	I-IIA	378	55.3	39	10.3	339	89.7		132	34.9	246	65.1	
	IIB-IVB	306	44.7	48	15.7	258	84.3	0.036	131	42.8	175	57.2	0.035
Histology	SCC	511	74.7	63	12.3	448	87.7		189	37.0	322	63.0	
	Non-SCC	173	25.3	24	13.9	149	86.1	0.6	74	42.8	99	57.2	0.18
Pelvic node metastasis	Negative	517	75.6	59	11.4	458	88.6		204	39.5	313	60.5	
	Positive	167	24.4	28	16.8	139	83.2	0.071	59	35.3	108	64.7	0.34
Tumor size (mm)	< 40	328	48.0	39	11.9	289	88.1		117	35.7	211	64.3	
	≥ 40	356	52.0	48	13.5	308	86.5	0.53	146	41.0	210	59.0	0.15
Treatment	Surgery	395	57.7	47	11.9	348	88.1		153	38.7	242	61.3	
	Others ^2^	289	42.3	40	13.8	249	86.2	0.45	110	38.1	179	61.9	0.86
PLR	< 131.44	421	61.5	21	5.0	400	95.0						
	≥ 131.44	263	28.5	66	33.5	197	66.5	< 0.001					
Platelet count (/μl)	< 350,000	597	87.3						197	33.0	400	67.0	
	≥350,000	87	12.7						66	75.9	21	24.1	< 0.001
Total		684	100	87	12.7	597	87.3		263	38.5	421	61.5	

SCC, squamous cell carcinoma; PLR, platelet-lymphocyte ratio.

^1^ A PLR cut-off value of 131.44 was employed in this analysis.

^2^ Concurrent chemoradiotherapy, radiotherapy and chemotherapy.

**Table 3:** Univariate and multivariate analyses for progression-free survival in cervical cancer patients

		Univariate analysis			Multivariate analysis ^1^			Multivariate analysis ^2^		
		Hazard ratio	95% CI	*p*-value	Hazard ratio	95% CI	*p*-value	Hazard ratio	95% CI	*p*-value
Age	< 50									
	≥ 50	1.3	0.99–1.71	0.058	1.12	0.82–1.55	0.47	1.06	0.78–1.45	0.70
Stage	I-IIA									
	IIB-IVB	3.87	2.94–5.17	< 0.001	2.16	1.48–3.17	< 0.001	2.16	1.48–3.16	< 0.001
Histology	SCC									
	Non-SCC	1.13	0.84–1.5	0.41	1.86	1.34–2.54	< 0.001	1.87	1.35–2.56	< 0.001
Pelvic node metastasis	Negative									
	Positive	2.61	2.01–3.38	< 0.001	1.91	1.45–2.51	< 0.001	2.00	1.52–2.64	< 0.001
Tumor size (mm)	< 40									
	≥ 40	4.22	3.63–7.09	< 0.001	2.66	1.84–3.89	< 0.001	2.65	1.83–3.86	< 0.001
Treatment	Surgery									
	Others ^3^	1.94	1.50–2.52	< 0.001	0.95	0.68–1.37	0.80	0.99	0.70–1.39	0.93
Platelet count (/μl)	< 350,000									
	≥350,000	1.90	1.35–2.62	< 0.001	1.63	1.14–2.28	0.0077			
PLR	< 125.23									
	≥ 125.23	1.39	1.07–1.79	0.012				1.31	1.01–1.70	0.044

SCC, squamous cell carcinoma; PLR, platelet-lymphocyte ratio; CI, confidence interval.

^1^ Multivariate analysis in which PLR is excluded from prognostic variables (platelet count is included).

^2^ Multivariate analysis in which platelet count is excluded from prognostic variables (PLR is included).

^3^ Concurrent chemoradiotherapy, radiotherapy and chemotherapy.

**Table 4:** Univariate and multivariate analyses for overall survival in cervical cancer patients

		Univariate analysis			Multivariate analysis ^1^			Multivariate analysis ^2^		
		Hazard ratio	95% CI	p-value	Hazard ratio	95% CI	*p*-value	Hazard ratio	95% CI	
Age	< 50									
	≥ 50	1.24	0.93–1.68	0.14	0.97	0.70–1.37	0.88	0.93	0.67–1.30	0.69
Stage	I-IIA									
	IIB-IVB	4.93	3.58–6.91	< 0.001	2.64	1.72–4.12	< 0.001	2.64	1.72–4.11	< 0.001
Histology	SCC									
	Non-SCC	1.14	0.82–1.55	0.43	2.09	1.46–2.94	< 0.001	2.05	1.44–2.88	< 0.001
Pelvic node metastasis	Negative									
	Positive	2.50	1.87–3.32	< 0.001	1.77	1.31–2.39	< 0.001	1.87	1.38–2.52	< 0.001
Tumor size (mm)	< 40									
	≥ 40	5.03	3.63–7.09	< 0.001	2.78	1.83–4.30	< 0.001	2.72	1.79–4.20	< 0.001
Treatment	Surgery									
	Others 3	2.26	1.70–3.01	< 0.001	1.12	0.77–1.63	0.56	1.15	0.80–1.67	0.45
Platelet count (/μl)	< 350,000									
	≥350,000	1.93	1.34–2.72	< 0.001	1.56	1.06–2.24	0.025			
PLR	< 131.44									
	≥ 131.44	1.59	1.20–2.11	0.0012				1.41	1.06–1.87	0.019

SCC, squamous cell carcinoma; PLR, platelet-lymphocyte ratio; CI, confidence interval.

^1^ Multivariate analysis in which PLR is excluded from prognostic variables (platelet count is included).

^2^ Multivariate analysis in which platelet count is excluded from prognostic variables (PLR is included).

^3^ Concurrent chemoradiotherapy, radiotherapy and chemotherapy.

In the present study, we first investigated the prognostic significance of elevated platelet counts and PLR in patients with FIGO stage IA-IVA cervical cancer. Then, we compared the clinical utilities of platelet counts and PLR for predicting the survival of patients with cervical cancer. Finally, we established a prognostic model using platelet counts and PLR to predict patient survival.

## RESULTS

### Prognostic significance of platelet counts

**Figure 1:**
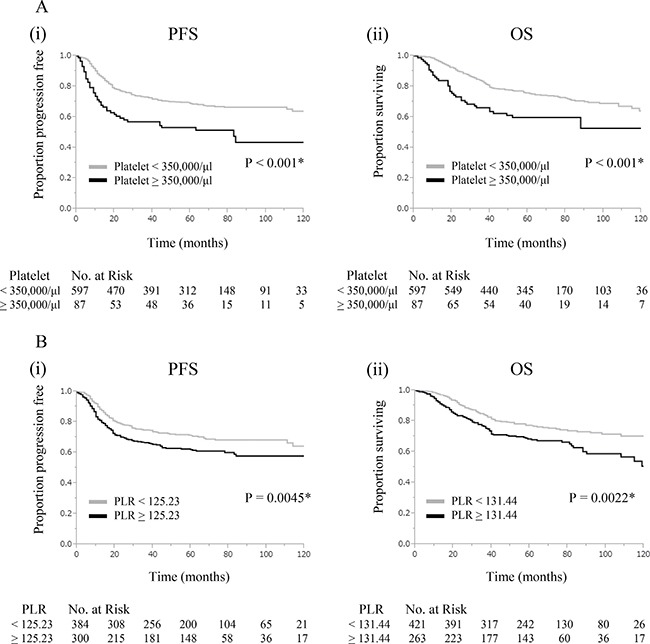
Clinical implications of platelet counts and PLR in cervical cancer patients (**A**) Significance of elevated platelet counts (Platelet count; ≥ 350,000/μl vs < 350,000/μl). (i) Kaplan-Meier estimates of progression-free survival. (ii) Kaplan-Meier estimates of overall survival. (**B**) Significance of elevated PLR. (i) Kaplan-Meier estimates of progression-free survival (PLR; ≥ 125.23 vs < 125.23). (ii) Kaplan-Meier estimates of overall survival (PLR; ≥ 131.44 vs < 131.44).

The clinicopathological characteristics of patients according to platelet counts are shown in Table 2. Among 684 patients, 87 (12.7%) had platelet counts equal to or greater than 350,000/ml (the thrombocytosis group) at the time of the initial diagnosis. Patients with thrombocytosis were significantly younger (*P <* 0.001) and presented with a more advanced clinical stage (*P* = 0.036) than those without thrombocytosis. Thrombocytosis correlated with significantly shorter PFS (*P <* 0.001) and OS (*P <* 0.001) in the univariate analysis and Kaplan-Meier analysis (Tables 3, 4, Figure 1A). In the multivariate analysis (Tables 3, 4), in addition to an advanced clinical stage, non-SCC histology, pelvic node metastasis, and larger tumor size, an elevated platelet count (> 350,000/μl) was found to be an independent prognostic factor of PFS (HR, 1.63; 95% CI, 1.14–2.28; *P* = 0.0077) and OS (HR, 1.56; 95% CI, 1.06–2.24; *P* = 0.025).

### Prognostic significance of PLR

ROC curves were described to select the optimal cut-off value for PLR (Supplementary Figure 1). The cut-off values of PLR for PFS and OS were 125.23 and 131.44, respectively. The clinicopathological characteristics of patients according to PLR are shown in Table 2 and Supplementary Table 1. Among 684 patients, 300 (43.9%) and 263 (38.5%) displayed PLR equal to or greater than 125.23 and 131.44, respectively. Patients with elevated PLR were significantly younger (*P* = 0.0059, *P* = 0.0013) and presented with a more advanced clinical stage (*P* = 0.014, *P* = 0.035) than those with normal PLR. As shown in Figure 1B, elevated PLR correlated with significantly shorter PFS and OS (PFS: *P* = 0.0045, OS: *P* = 0.0022). In the multivariate analysis, in addition to an advanced clinical stage, non-SCC histology, pelvic node metastasis, and larger tumor size, elevated PLR remained an independent prognostic factor of PFS (Table 3: HR, 1.31; 95% CI, 1.01–1.70; *P* = 0.044) and OS (Table 4: HR, 1.41; 95% CI, 1.06–1.87; *P* = 0.019).

### Comparison of utilities of platelet counts versus PLR

**Figure 2:**
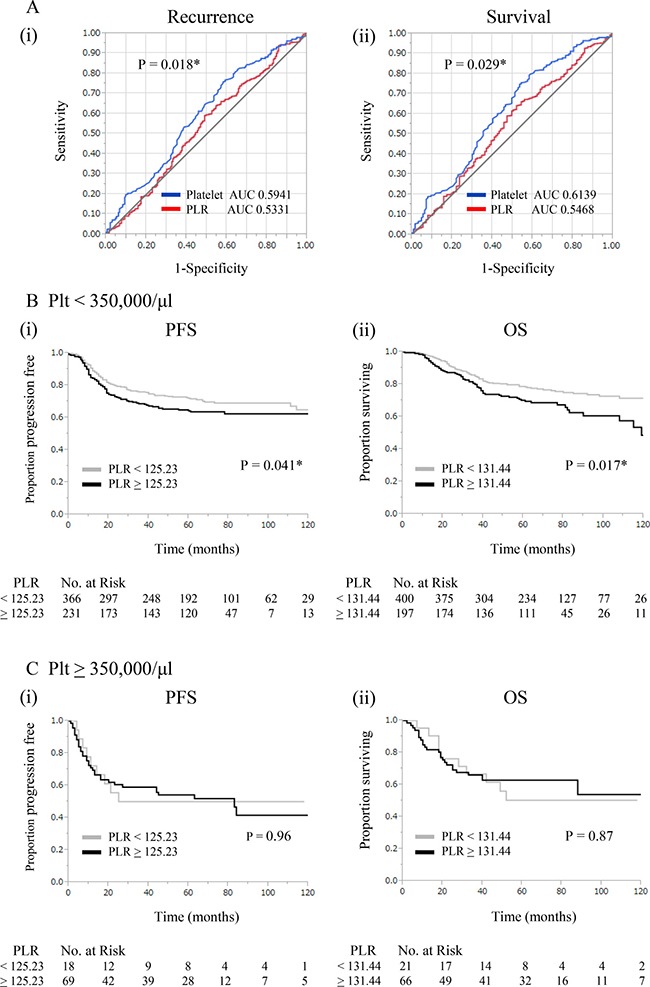
Comparison of clinical utilities of platelet counts and platelet-lymphocyte ratios (**A**) ROC curves for (i) recurrence and (ii) survival at 3 years for platelet counts and PLR. (**B**) Significance of PLR in patients without thrombocytosis (< 350,000/μl). (i) Kaplan-Meier estimates of progression-free survival (PLR; ≥ 125.23 vs < 125.23). (ii) Kaplan-Meier estimates of overall survival (PLR; ≥ 131.44 vs < 131.44). (**C**) Significance of PLR in patients with thrombocytosis (≥ 350,000/μl). (i) Kaplan-Meier estimates of progression-free survival (PLR; ≥ 125.23 vs < 125.23). (ii) Kaplan-Meier estimates of overall survival (PLR; ≥ 131.44 vs < 131.44).

In order to compare the clinical utilities of platelet counts and PLR for predicting patient prognoses, ROC curves for platelet counts and PLR were generated and compared (Figure 2A). The area under the ROC curve (AUC) for predicting recurrence using platelet counts and PLR were 0.5941 (95% CI, 0.5448–0.6415) and 0.5331 (95% CI, 0.4833–0.5822), respectively. The AUC for predicting survival using platelet counts and PLR were 0.6139 (95% CI, 0.5552–0.6695) and 0.5468 (95% CI, 0.4889–0.6034), respectively. Platelet counts showed significantly greater AUC values than PLR for predicting recurrence (*p* = 0.018) and survival (*p* = 0.029).

### Prognostic models using platelet counts and PLR

In order to establish a model for the prediction of life expectancy, PFS and OS were first assessed according to platelet counts and PLR. As shown in Figure 2B, in patients without thrombocytosis, elevated PLR correlated with shorter PFS (*P* = 0.041) and OS (*P* = 0.017). In contrast, in patients with thrombocytosis, survival was not influenced by PLR, indicating that it does not provide any prognostic information in this patient population (Figure 2C).

**Figure 3:**
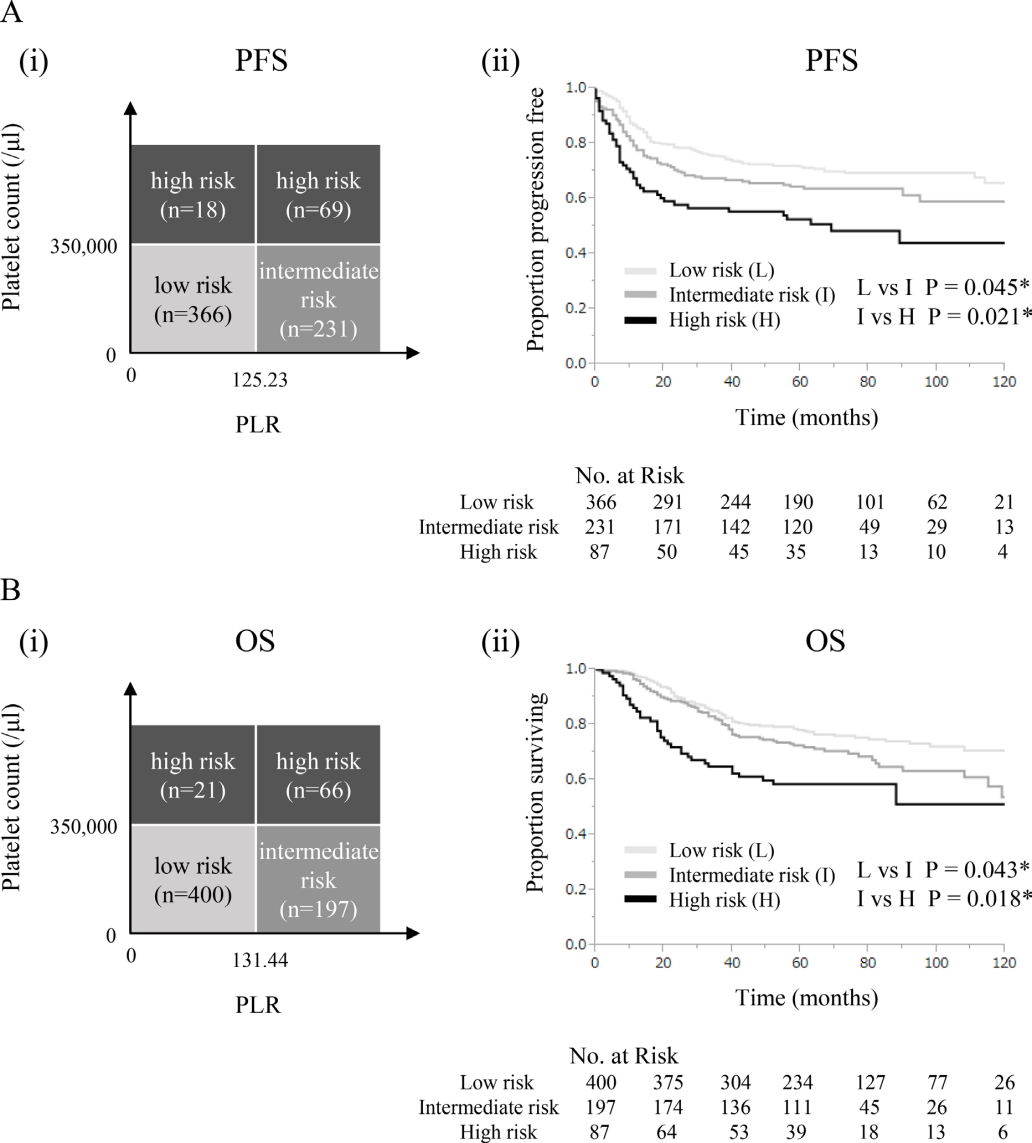
Prognostic model using platelet counts and PLR (**A**) (i) Risk classification for progression-free survival. (ii) Kaplan-Meier estimates of progression-free survival based on risk classification. (**B**) (i) Risk classification for overall survival. (ii) Kaplan-Meier estimates of overall survival based on risk classification.

Based on these results, we finally established a prognostic model in which patients were divided into 3 prognostic groups (Figure 3A(i), 3B(i)): high-risk (patients with thrombocytosis regardless of PLR); intermediate-risk (patients with elevated PLR without thrombocytosis); low-risk (none of the above). As shown in Supplementary Table 2, differential treatment outcomes were observed in association with the risk classifications. When PFS and OS were compared between the groups, patients in the high-risk group showed significantly lower PFS and OS rates than those in the intermediate-risk group (Figure 3A (ii): *P* = 0.021, 3B (ii): *P* = 0.018). Moreover, the PFS and OS rates of patients in the intermediate-risk group were significantly lower than those in the low-risk group (Figure 3A (ii): *P* = 0.045, 3B (ii): *P* = 0.043).

## DISCUSSION

In the present study, we showed that an elevated platelet count (≥ 350,000/μl) was an independent predictor of shorter PFS and OS in cervical cancer patients. These results are consistent with previous findings (Table 1). Among our study population, 12.7% of patients displayed elevated platelet counts (≥ 350,000/μl), which correlated with a younger age and advanced clinical stage. We also observed elevated PLR (≥ 125.23 for PFS and ≥ 131.44 for OS) in approximately 40% of patients, and identified it as an independent predictor of shorter PFS and OS in cervical cancer patients. This result is also consistent with previous findings (Table 1). However, since most of the studies that previously investigated the significance of increased PLR only included surgically-treated early-stage cervical cancer patients (Table 1), the present study provides a novel insight into PLR in cervical cancer treatment: elevated PLR at the initial diagnosis is prognostically important regardless of the clinical stage and treatment modality. Moreover, our ROC analysis demonstrated, for the first time, that platelet counts are significantly superior to PLR for the prediction of patient prognoses. We consider this result to be clinically important because it suggests that platelet counts need to be preferentially examined in patients with cervical cancer.

There are currently no universally accepted risk classifications that may be applied to all cervical cancer patients: i.e. patients treated with surgery, definitive radiotherapy, and chemotherapy. Thus, the results of the present study may have valuable clinical implications. Since the present study includes stage IA-IVA cervical cancer patients treated with surgery, definitive radiotherapy, or chemotherapy, the prognostic model proposed herein may be applied to all cervical cancer patients. Moreover, our prognostic model requires only low-cost peripheral blood examinations to identify a group of patients at high risk of recurrence. As shown in Figure 3, we demonstrated that it was possible to divide patients into 3 prognostic groups using platelet counts and PLR: high-risk (patients with thrombocytosis regardless of PLR); intermediate-risk (patients with elevated PLR without thrombocytosis); low-risk (none of the above). This prognostic model may have advantages that are relevant to clinical practices: this simple model offers individualized survival estimates (Figure 3A (ii), 3B (ii)). In addition, this model may enable physicians to offer closer follow-ups for patients in the intermediate- and high-risk groups. The results shown in Figures 2, 3 also provide important information on the clinical applications of platelet counts and PLR: we recommend that platelet counts be initially examined for survival estimations in cervical cancer patients. PLR may then be evaluated in patients without thrombocytosis only because it did not provide prognostic information on patients with thrombocytosis.

Based on the poor prognosis of cervical cancer patients who display elevated platelet counts or PLR, novel treatment strategies need to be developed. The mechanisms responsible for increased platelet production in cervical cancer and subsequent increases in the aggressiveness of the disease remain poorly understood. However, theoretically, treatments targeting thrombopoiesis-stimulating cytokines or growth factors, their receptors, or their downstream effectors may exhibit therapeutic efficacy in cervical cancer patients displaying pretreatment thrombocytosis. In a previous study, the inhibition of thrombopoietin or IL-6 prevented the development of thrombocytosis in mice and significantly enhanced the therapeutic efficacy of paclitaxel in mouse models of epithelial ovarian cancer [24]. Thus, in order to obtain a clearer understanding of platelet count alterations and advance the development of novel treatments, further mechanistic investigations on cervical cancer are warranted.

The limitations of our study need to be addressed. The first limitation is that the present study was conducted at a single institution. We intend to verify our clinical findings in collaborative multi-institutional studies in the future. Another limitation is the retrospective nature of the present study. The significance of elevated platelet counts, PLR, and our prognostic model consisting of platelet counts and PLR need to be prospectively evaluated in future studies. The second limitation is the cut-off values used for thrombocytosis. In the present study, we defined elevated platelet counts as greater than or equal to 350,000/μl. The cut-off values for thrombocytosis in previous studies that investigate the significance of thrombocytosis in cervical cancer ranged between 200,000/μl and 400,000/μl, with 400,000/μl being the most popular cut-off value (Table 1). However, most of the studies listed in Table 1 were from countries other than Japan. In studies on various malignant tumors from Japanese institutions, the cut-off values for thrombocytosis were lower: most studies employed a cut-off value between 22,000/μl and 370,000/μl to define thrombocytosis [23, 25–29]. The reason why a lower cut-off value was employed in studies from Japanese institutions currently remains unknown; however, the baseline platelet count in cancer patients may differ due to ethnicity. We also showed that patients with thrombocytosis were significantly younger (*P* < 0.001) than those without thrombocytosis. This result is consistent with a recent finding on cervical cancer [17]. However, the reason for this phenomenon remains unknown. Thus, the optimal platelet threshold for diagnosing thrombocytosis and the underlying mechanisms responsible for the development of thrombocytosis need to be investigated in future studies.

In conclusion, thrombocytosis and elevated PLR at the time of the initial diagnosis were identified as independent predictors of PFS and OS in FIGO stage IA-IVA cervical cancer patients. Platelet counts were significantly superior to PLR for predicting patient prognoses. Our proposed prognostic model consisting of platelet counts and PLR offers individualized and accurate survival estimates.

## MATERIALS AND METHODS

### Patients

Permission to proceed with data acquisition and analyses was obtained from the Institutional Review Board of Osaka University Hospital. A list of patients diagnosed with FIGO stage IA-IVA cervical cancer and treated at Osaka University Hospital between November 1993 and December 2011 was generated from our institutional tumor registry, and their clinical data were retrospectively analyzed. Patients who had been diagnosed with other types of cancers within the past 5 years, had a history of splenectomy, myeloproliferative disorders, or acute inflammatory disease were excluded. Of the 684 patients included in the present study, 286 had been examined in a previous clinical study [13].

### Treatment and post-treatment follow-up

Patients were treated in accordance with institutional treatment guidelines. Briefly, patients with FIGO stage IA2-IIB cervical cancer and younger than 70 years were treated with radical hysterectomy plus pelvic lymphadenectomy with or without adjuvant radiotherapy as described previously [30]. Adjuvant radiotherapy with or without platinum-based concurrent chemotherapy, was indicated when a patient's pathological report displayed any one of the following ‘high-risk’ prognostic factors: parametrial invasion, pelvic lymph node metastasis, or a positive surgical margin, or one of the following ‘intermediate-risk’ prognostic factors: deep stromal invasion, lymphovascular space invasion, or a large tumor (more than 4 cm in diameter), as reported previously [30]. Patients with FIGO stage III-IV disease, patients with FIGO stage I-II disease and older than 70 years, or patients with FIGO stage IA2-IIB disease and younger than 70 years who desired definitive radiotherapy rather than surgery were treated with definitive radiotherapy consisting of external beam radiation therapy followed by high-dose-rate intracavitary brachytherapy with or without platinum-based concurrent chemotherapy as described previously [31]. Patients with systemic disease were primarily treated with platinum-based chemotherapy as described previously [32, 33]. Follow-up examinations performed after the initial treatment were conducted by gynecological oncologists or/and radiation oncologists at regular intervals in an outpatient clinic, as reported previously [31, 34].

### Definition of elevated platelet counts and PLR

During the period between the first presentation and the start day of the initial treatment, all patients underwent at least 2 blood tests including complete blood counts. Thrombocytosis was defined as platelet counts equal to or greater than 350,000/μl on at least 2 separate occasions, as described previously [13]. Elevated PLR for predicting progression-free survival (PFS) or overall survival (OS) were defined as PLR equal to or greater than 125.23 or 131.44, respectively (Supplementary Figure 1). The cut-off values for PLR were defined based on the maximum Youden index (i.e. sensitivity+specificity-1) in the time-dependent receiver operating characteristic (ROC) curve for PFS and OS, as reported previously [35, 36].

### Statistical analysis

PFS was defined as the time from the date of therapy to the date of the first physical or radiographical evidence of disease progression. OS was defined as the time from the date of therapy to the date of death.

Time-dependent ROC curves were generated to evaluate the diagnostic performance of platelet counts and PLR for predicting recurrence or death at 3 years after the treatment. Differences in AUCs were analyzed according to the methods described in a previous study [37].

Continuous data were compared between the groups using the Student's *t*-test or Log-rank test, where appropriate. Frequency counts and proportions were compared between the groups using the chi-squared test or a two-tailed Fisher's exact test, where appropriate. The survival analysis was based on the Kaplan-Meier method and was compared by the Wilcoxon test. Cox's proportional hazards regression analysis was performed to identify significant independent prognostic factors for survival. *P*-values of < 0.05 were considered to be significant. All analyses were performed using the software JMP Pro version 11.0 (SAS Institute, Cary, NC).

## SUPPLEMENTARY MATERIALS FIGURES AND TABLES


