# Supplementary material for: Comparison of clinical utilities of the platelet count and platelet-lymphocyte ratio for predicting survival in patients with cervical cancer: a single institutional study and literature review
Source: Oncotarget. 2017 Jul 25;8(33):55394–404. doi: 10.18632/oncotarget.19560 (PMC5589667; doi:10.18632/oncotarget.19560)
Supplement: Supplementary file 1 [file oncotarget-08-55394-s001.pdf]

Comparison of clinical utilities of the platelet count and platelet-lymphocyte ratio for predicting survival in patients with cervical cancer: a single institutional study and literature review

SUPPLEMENTARY MATERIALS

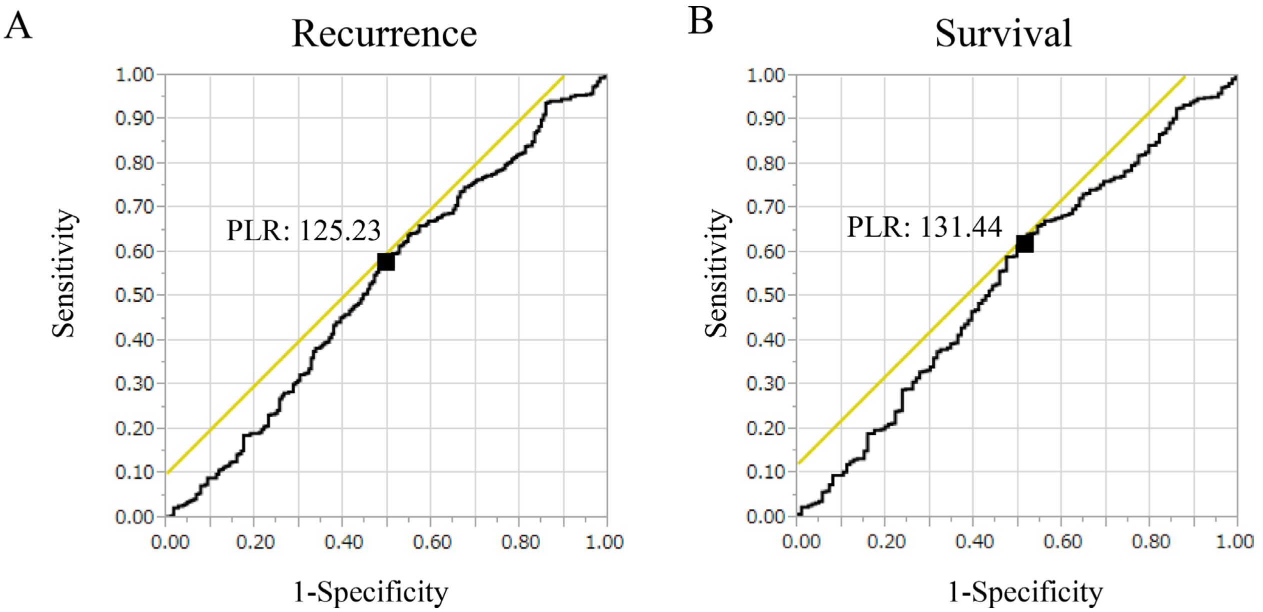

Supplementary Figure 1: ROC curves for (A) recurrence and (B) survival at 3 years.

Supplementary Table 1: Clinicopathological characteristics of patients according to PLR [1]

		All patients		Elevated PLR		Normal PLR		
		No	(%)	No	(%)	No	(%)	*p*-value
Age	< 50	268	39.2	135	50.4	133	49.6	
	≥ 50	416	60.8	165	39.7	251	60.3	0.0059
Stage	I-IIA	378	55.3	150	39.7	228	60.3	
	IIB-IVB	306	44.7	150	49.0	156	51.0	0.014
Histology	SCC	511	74.7	221	43.2	290	56.8	
	Non-SCC	173	25.3	79	45.7	94	54.3	0.58
Pelvic node metastasis	Negative	517	75.6	233	45.1	284	54.9	
	Positive	167	24.4	67	40.1	100	59.9	0.26
Tumor size (mm)	< 40	328	48.0	137	41.8	191	58.2	
	≥ 40	356	52.0	163	45.8	193	54.2	0.29
Treatment	Surgery	395	57.7	171	43.3	224	56.7	
	Others 2	289	42.3	129	44.6	160	55.4	0.73
Platelet count (/µl)	< 350,000	597	87.3	231	38.7	366	61.3	
	≥ 350,000	87	12.7	69	79.3	18	20.7	< 0.001
Total		684	100	300	43.9	384	56.1	

SCC, squamous cell carcinoma; PLR, platelet-lymphocyte ratio.
[1]A PLR cut-off value of 125.23 was employed in this analysis.
[2]Concurrent chemoradiotherapy, radiotherapy and chemotherapy.

Supplementary Table 2: Treatment outcomes according to the risk classification

	Cut-off value of PLR employed	Low-risk group	Intermediate-risk group	High-risk group	*p*-value
3-year recurrence rate, % (N/Total)	125.23	25.5% (89/349)	33.2% (73/220)	44.0% (37/84)	0.0023
3-year death rate, % (N/Total)	131.44	15.7% (60/383)	21.0% (39/186)	35.3% (30/85)	< 0.001

PLR, platelet-lymphocyte ratio.
